# First human case report of Crohn's disease with coexistent acute appendicitis treated by endoscopic retrograde appendicitis therapy

**DOI:** 10.3389/fmed.2023.1171463

**Published:** 2023-06-09

**Authors:** Guang-xing Cui, Muhammad Zulqarnain, Qi-feng Lou, Hong-zhang Shen, Wen Lyu, Xia Wang, Haitao Huang, Hamse Mohamoud Abdi, Lingfei Gu, Shijie Fang, Fangzhou Liu, Liqian Ling, Yu Huang, Linglin Chu, Saboor Saeed

**Affiliations:** ^1^Department of Gastroenterology, Affiliated Hangzhou First People's Hospital, Zhejiang University School of Medicine, Hangzhou, China; ^2^Key Laboratory of Integrated Traditional Chinese and Western Medicine for Biliary and Pancreatic Diseases of Zhejiang Province, Hangzhou, China; ^3^Hangzhou Institute of Digestive Diseases, Hangzhou, China; ^4^Department of Gastroenterology, Fourth Clinical Medical College of Zhejiang Chinese Medical University, Hangzhou, China

**Keywords:** Crohn's disease, acute appendicitis, endoscopic retrograde appendicitis therapy, endoscopic retrograde appendicography, coexistent

## Abstract

**Background:**

The coexistence of Crohn's disease (CD) and acute appendicitis (AA) is rare. In this situation, therapeutic experience is lacking and the strategy is paradoxical and intractable. Appendectomy is the gold standard for the treatment of AA whereas a nonsurgical approach is recommended for CD.

**Case summary:**

A 17-year-old boy was hospitalized for right lower abdominal pain with fever of 3 days. He had the CD for 8 years. Two years ago, he underwent surgery for anal fistula with the complication of CD. His temperature was elevated at 38.3°C at admission. On physical examination, there was McBurney tenderness with mild rebound tenderness. Abdominal ultrasonography showed that the appendix was notably enlarged and dilated at 6.34 cm long and 2.76 cm wide. These findings were suggestive of uncomplicated AA in this patient with active CD. Endoscopic retrograde appendicitis therapy (ERAT) was performed. The patient had complete pain relief immediately after the procedure without tenderness in the right lower abdomen. During 18 mo follow-up, he had no more attacks in his right lower abdomen.

**Conclusion:**

ERAT was effective and safe in a CD patient with coexisting AA. Such cases can avoid surgery and its-related complications.

## Introduction

Acute appendicitis (AA) is one of the most common abdominal emergencies worldwide, and affects 5.7–57/per 100 000 individuals each year with the highest incidence rate in children and adults (1). Appendectomy is still the gold standard for management of uncomplicated acute appendicitis (UAA) (1, 2). However, the surgical complications should not be neglected, with an overall incidence rate of 20.6%, including minor and major complications in open and laparoscopic appendectomies (3).

Recently, more research has focused on the efficacy and safety of conservative treatment for UAA. Several systematic reviews and meta-analyses have concluded most UAA can be managed with an antibiotic-first approach (4, 5). However, they also pointed out that the recurrence rate of appendicitis reached 19.2–22.6% within the first year. Besides, according to the 2020 WSES Jerusalem guidelines, the recurrence rate is as high as 39% after 5 years' follow-up, indicating that conservative treatment is not suitable for patients with appendicolith (1).

Crohn's disease (CD) is an autoimmune disease that is characterized by a transmural inflammatory reaction. It can cause inflammation of the digestive tract, which leads to fistula, abdominal pain, severe diarrhea, fatigue, weight loss and malnutrition (6, 7). Coexistence of CD and AA is uncommon (8, 9). In this situation, therapeutic experience is lacking and the treatment strategy is paradoxical and intractable. The result of conservative therapy for AA may be totally different, whereas a nonsurgical approach is reasonable for CD.

Endoscopic retrograde appendicitis therapy (ERAT) is a novelty endoscopic minimally invasive procedure for diagnosis and management of AA, which was firstly reported by Liu et al. (10). AA can be cured by ERAT without a need for surgery. Here, we report an adolescent case of active CD with coexisting UAA that was successfully managed by ERAT. To the best of our knowledge, this is the first human case of ERAT in a patient with coexistence of CD and AA.

## Case presentation

### Chief complaints

A 17-year-old boy was hospitalized due to right lower abdominal pain with fever for 3 days.

### History of present illness

Local hospital computed tomography (CT) showed thickened ileocecal intestinal wall. Colonoscopy displayed mucosal changes in CD and pathological analysis confirmed active CD. For further diagnosis and treatment, he came to our hospital. We arranged an abdominal CT scan, which showed ileocecal intestinal wall thickening. Abdominal ultrasonography detected an obviously dilated and longer appendix. A preliminary diagnosis of UAA was established. The patient had indications for colonoscopy and ERAT. After obtaining informed consent from his parents, the procedure was performed.

### History of past illness

He had CD for 8 years. Two years ago, he underwent surgery for anal fistula with the complication of CD.

### Physical examination

Body temperature was elevated to 38.3°C admission. On physical examination, there was McBurney tenderness with mild rebound tenderness.

### Laboratory examinations

Blood tests after admission showed that white blood cell count was 13.8 × 10^9^/L, hemoglobin was 111 g/L, C-reactive protein was 89 mg/L (normal value < 8 mg/L), neutrophil percentage was 81.5%, and serum procalcitonin was 0.55 ng/ml (normal value < 0.5 ng/ml).

### Imaging examinations

**Figure 1:**
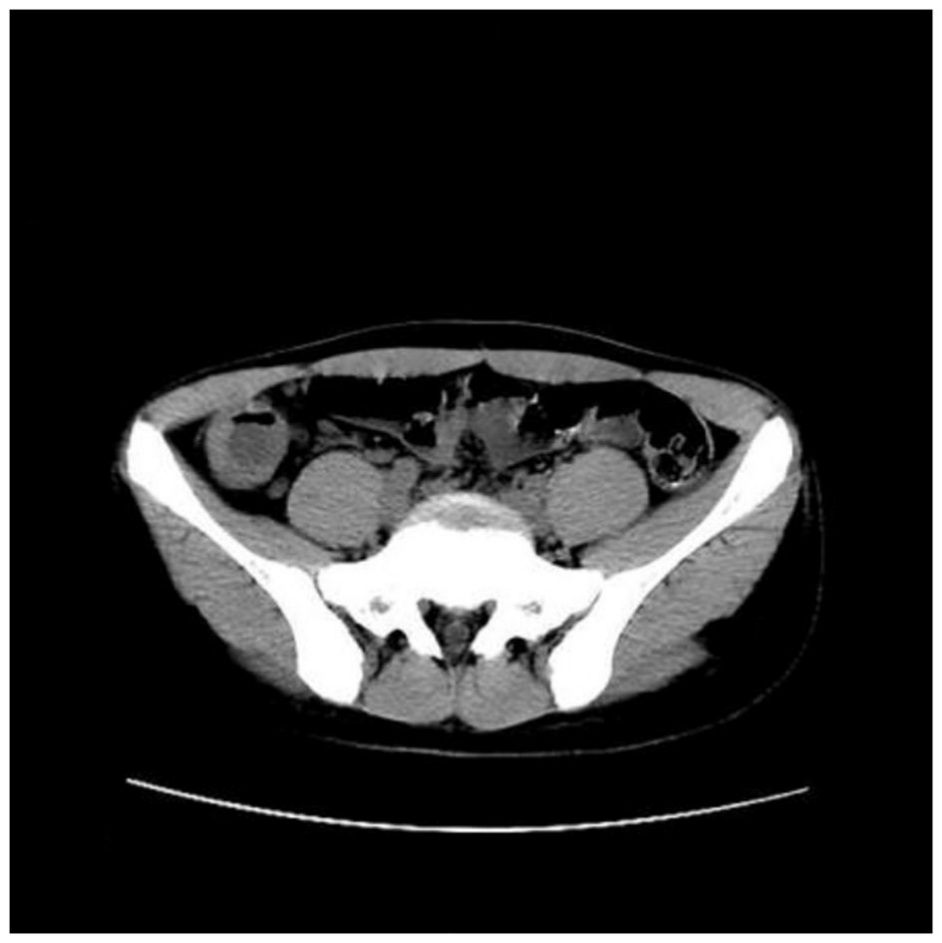
Abdominal CT scan showed obvious thickening of the ileocecal wall.

**Figure 2:**
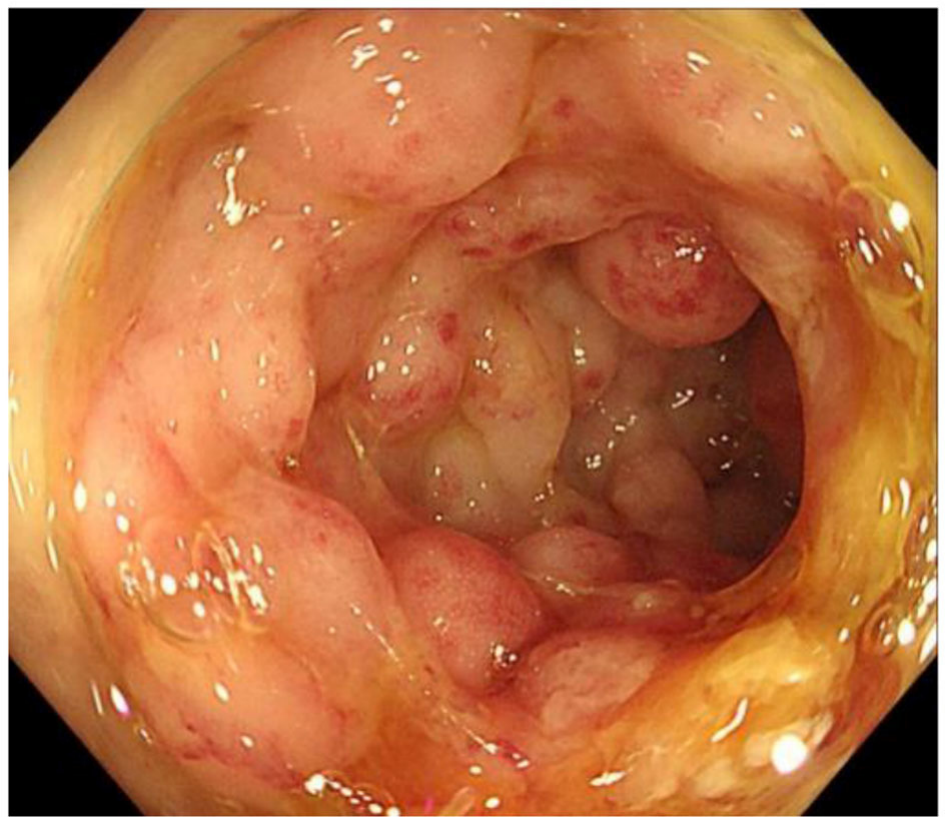
Colonoscopic images of the inferior ascending colon showing circumferential granular hyperplasia, polypoid mucosal protrusion and cobblestone-like changes.

Abdominal CT scan showed obvious thickening of the ileocecal wall (Figure 1). Abdominal ultrasonography showed that the appendix was notably enlarged and dilated at 6.34 cm long and 2.76 cm wide. Colonoscopy revealed that the terminal ileum, ileocecal valve, and inferior ascending colon showed circumferential granular hyperplasia and polypoid mucosal protrusion, with cobblestone-like changes and a fragile, local, longitudinal deep ulcer that easily bled, accompanied by mild stenosis in the cecum and ascending colon (Figure 2).

## Final diagnosis

The final diagnosis was UAA in a patient with active CD.

## Treatment

**Figure 3:**
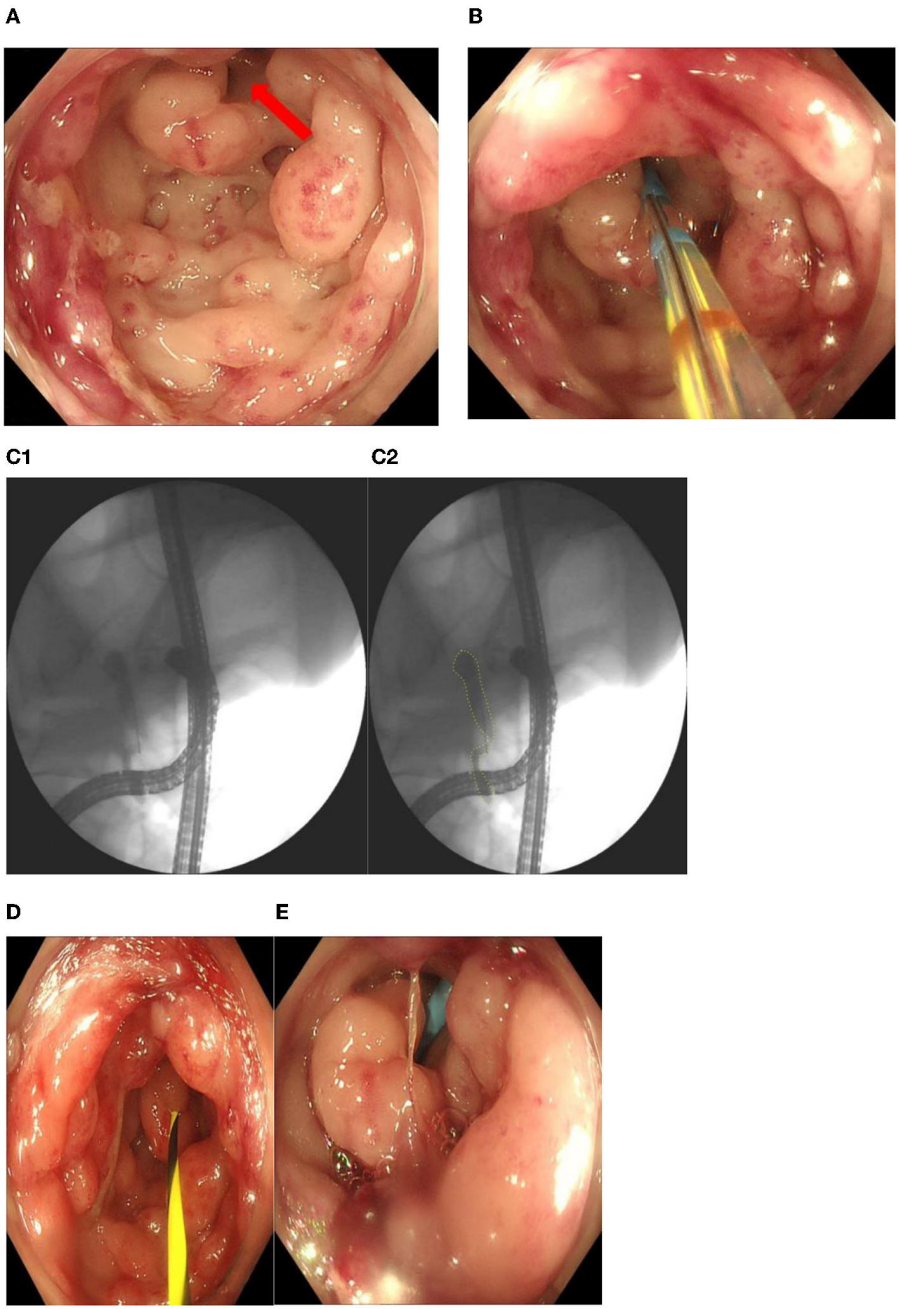
**(A)** The real orifice of appendix at the 12 o'clock position (red arrow). **(B)** Cannulation with an endoscopicretrograde cholangiopancreatography sphincterotome. **(C1, C2)** Under fluoroscopy, the appendiceal lumen was clearly displayed, which had a length of 10.0 cm and the appendiceal inner diameter was entirely dilated to 1.2 cm in the upper half and 0.5–0.6 cm in the lower half. **(D, E)** A pigtail plastic 6 Fr stent of 5-cm length was placed into the lumen by a guidewire for postoperative drainage.

ERAT was performed. The cecal mucosa was rough and hyperproliferated, so it was difficult to detect the appendiceal orifice. After careful detection, at the 12 o'clock position, we found the real orifice (Figure 3A). While it was successfully cannulated with an endoscopic retrograde cholangiopancreatography sphincterotome, a lot of pus was exuding from the appendix (Figure 3B). Under fluoroscopy, the appendiceal lumen was clearly displayed, which had a length of 10.0 cm and the appendiceal inner diameter was entirely dilated to 1.2 cm in the upper half and 0.5–0.6 cm in the lower half (Figures 3C1, C2). However, no obvious sign of appendicolith was found from fluoroscopy. Then the lumen was irrigated with metronidazole, during which there was continuous pus flow. After decompression and clearance of the lumen, a pigtail plastic 6 Fr stent of 5-cm length was successfully placed into the lumen by a guidewire for postoperative drainage (Figures 3D, E).

## Outcome and follow-up

The patient had complete pain relief immediately after the procedure without tenderness in the right lower abdomen. He recovered uneventfully and was discharged on postoperative day 3. Two months later, a second colonoscopy in our endoscopy center was performed, showing no stent in the appendix, during which fecal microbiota transplantation was conducted to achieve better recovery of CD. One year later, a third colonoscopy showed satisfactory recovery of CD. Ultrasound and CT scan showed no abnormality in the appendix. During a total of 18 mo follow-up, he had no more attacks in his right lower abdomen.

## Discussion

CD is an autoimmune disease that affects the gastrointestinal tract, and is accompanied by parenteral manifestations and related immune disorders. For patients with CD, nonsurgical therapy is commonly accepted as a first approach (6, 7). The application of surgery in CD patients is limited, unless CD is complicated with severe intestinal stenosis or fistula. Surgery may lead to potentially severe complications, higher recurrence rate, and decreased quality of life (9, 11). Appendectomy in patients with CD is associated with a high risk of fistula. Crohn stated in 1949, “My own experience does not favor appendectomy at the time of exploration, because such a procedure is unnecessarily superfluous and tends to fistula formation” (12). A retrospective study by Fonkalsrud et al. (13) indicated that in nine young patients with CD who had undergone previous appendectomies, seven had subsequent enteric fistula and/or obstruction involving the cecum.

Coexistence of CD and AA is difficult because the optimal therapeutic strategy is unclear. Patients with CD need to avoid surgery as far as possible. However, a patient with UAA needs to be managed by appendectomy. Fortunately, the two conditions rarely coexist (8, 9). The mechanism for coexistence remains unknown. One hypothesis is that it is associated with the chemotactic gradient of neutrophil migration. In CD, the chemotactic gradient of inflamed tissue may be stronger than that from an inflamed appendix, which attracts most neutrophils away from the appendix. Undoubtedly, it is an intractable situation if such a coexistence develops, because there is lack of therapeutic experience.

A novel approach of ERAT for endoscopically managing AA was reported by Liu et al. (10). It is a minimally invasive procedure to diagnose and manage AA with the aim of preserving the appendix and its functions. A recent systematic review and meta-analysis showed that ERAT is effective and safe for AA, with a clinical efficacy rate of 99.26% and recurrence rate of 6.01% (14). Compared with laparoscopic and open appendectomy, ERAT has several advantages. Ding et al. (15) reported in the ERAT group that the length of hospital stay, surgery-related complications, and inpatient expenses were all significantly less than for either type of appendectomy. A recent open-label randomized trial from China showed that there were no significant differences in clinical success and adverse events between ERAT and appendectomy (16).

ERAT has an important diagnostic function. CD and AA may both present with pain in the ileocecal area, which leads to difficulty in distinguishing them preoperatively. Few previous studies have focused on the preoperative laboratory findings, including hemoglobin level, white blood cell count, and mean corpuscular volume count, with unsatisfactory sensitivity and specificity (9, 17). However, using an ERAT technique, the appendiceal orifice can be observed clearly and endoscopic retrograde appendicography after cannulation can detect whether there is any abnormality such as fecalith or dilatation or stricture in the appendiceal lumen. According to research by Li et al. (18), the diagnostic accuracy for AA with combination of colonoscopy and endoscopic retrograde appendicography is as high as 95.2%. In the present study, colonoscopy revealed that the appendiceal orifice showed inflammation with pus covering it. Appendicography showed that the whole appendiceal lumen had severe dilatation, indicating high luminal pressure. A lot of milk-like pus was seen exuding from the lumen. The diagnosis of AA was accurate. The appendiceal lumen was completely cleaned with antibiotics and a stent was placed for postoperative drainage. To the best of our knowledge, this is the first human case of ERAT to manage coexistence of CD and AA.

This case shows that ERAT has unique advantages for patients with coexistence of CD and AA, as follows: (1) AA can be cured completely by such a super-minimally invasive procedure; (2) the appendix together with its functions can be preserved; (3) ERAT can avoid surgery-related risks in patients with CD; and (4) ERAT decreases the rate of negative appendectomy and improves diagnostic accuracy of endoscopic retrograde appendicography. However, like any other surgery or procedure, ERAT technique also has its disadvantages and potential risks of complications. (1) bowl preparation: before ERAT, most patients with appendicitis can tolerate bowl preparation by drinking plenty of water, but a few are intolerable. Besides, bad bowl preparation will affect the procedure, even resulting in failure. (2) missing diagnosis of appendiceal tumor: although the diagnostic accuracy for AA with combination of colonoscopy and endoscopic retrograde appendicography seem to be satisfying, it still has potential risk of missing early or concealed appendix tumors. (3) deficiency of ERAT's accessory instruments: currently, ERCP's stone retrieval balloon is used in patients with appendicolith. However, sometimes, the balloon seems to be too large to remove fecalith. (4) complications: bleeding and perforation may occur during cannulation of appendix, others rare complication like stenting migration into the appendiceal lumen also had been reported (19).

## Conclusion

Our case showed that ERAT was effective and safe for managing a patient with coexisting CD and AA. Such cases can avoid surgery and its-related complications. Endoscopic retrograde appendicography is helpful to distinguish AA from CD. More cases need to be accumulated to further prove its efficacy and safety.

## Core tip

Coexistence of Crohn's disease (CD) and acute appendicitis (AA) is rare. In this situation, therapeutic experience is lacking and the treatment strategy is controversial. Surgery is the preferred treatment for AA whereas non-surgical approach is often recommended for CD. We successfully treated a 17-year-old boy with coexistent CD and AA using the innovative procedure of endoscopic retrograde appendicitis therapy for AA. The boy was recovered rapidly without need for appendectomy.

## Data availability statement

The original contributions presented in the study are included in the article/supplementary material, further inquiries can be directed to the corresponding author.

## Ethics statement

The studies involving human participants were reviewed and approved by Hangzhou First People's Hospital. Written informed consent to participate in this study was provided by the participants' legal guardian/next of kin. Written informed consent was obtained from the individual(s) and minor(s)' legal guardian/next of kin, for the publication of any potentially identifiable images or data included in this article.

## Author contributions

WL is an advanced endoscopist who performed the ERAT procedure. G-xC and MZ drafted the manuscript. Q-fL, H-zS, XW, and HH analyzed the special part of the case. HM, LG, SF, FL, LL, and YH collected the data. LC and SS revised the manuscript. All authors contributed to the article and approved the submitted version.
